# Measuring relative vibrotactile spatial acuity: effects of tactor type, anchor points and tactile anisotropy

**DOI:** 10.1007/s00221-018-5387-z

**Published:** 2018-10-06

**Authors:** Rebekka Hoffmann, Vigdís Vala Valgeirsdóttir, Ómar I. Jóhannesson, Runar Unnthorsson, Árni Kristjánsson

**Affiliations:** 1 Faculty of Psychology, School of Health Sciences, University of Iceland, Reykjavik, Iceland; 2 Faculty of Industrial Engineering, Mechanical Engineering and Computer Science, University of Iceland, Reykjavik, Iceland

**Keywords:** Tactile spatial acuity, Vibrotactile, Tactor type, Tactile anisotropy, Inter-tactor distance, Relative point localization, Spine, Anchor point, Body midline

## Abstract

Vibrotactile displays can compensate for the loss of sensory function of people with permanent or temporary deficiencies in vision, hearing, or balance, and can augment the immersive experience in virtual environments for entertainment, or professional training. This wide range of potential applications highlights the need for research on the basic psychophysics of mechanisms underlying human vibrotactile perception. One key consideration when designing tactile displays is determining the minimal possible spacing between tactile motors (tactors), by empirically assessing the maximal throughput of the skin, or, in other words, vibrotactile spatial acuity. Notably, such estimates may vary by tactor type. We assessed vibrotactile spatial acuity in the lower thoracic region for three different tactor types, each mounted in a 4 × 4 array with center-to-center inter-tactor distances of 25 mm, 20 mm, and 10 mm. Seventeen participants performed a relative three-alternative forced-choice point localization task with successive tactor activation for both vertical and horizontal stimulus presentation. The results demonstrate that specific tactor characteristics (frequency, acceleration, contact area) significantly affect spatial acuity measurements, highlighting that the results of spatial acuity measurements may only apply to the specific tactors tested. Furthermore, our results reveal an anisotropy in vibrotactile perception, with higher spatial acuity for horizontal than for vertical stimulus presentation. The findings allow better understanding of vibrotactile spatial acuity and can be used for formulating guidelines for the design of tactile displays, such as regarding inter-tactor spacing, choice of tactor type, and direction of stimulus presentation.

## Introduction

Vibrotactile devices deploying mechanical stimulation through tactile motors (tactors) in combination with a sophisticated haptic language are powerful tools with a wide range of applications. As parts of sensory substitution devices (SSDs), they can compensate for the loss of sensory function and augment sensory experiences of people with permanent (visually- or hearing impaired) or temporary deficiencies (e.g., rescue teams in difficult environments) (Bach-y-Rita and Kercel 2003; Cosgun et al. 2014; Hoffmann et al. 2018; Kristjánsson et al. 2016). Furthermore, vibrotactile arrays worn around the waist can assist people with balance impairments by providing vibratory feedback (Wall and Weinberg 2003) and can enhance immersive experiences in virtual environments for entertainment, or professional training (Faroque et al. 2015; Guinan et al. 2012). In the last decade, vibrotactile equipment has become more available, affordable, and less intrusive (Choi and Kuchenbecker 2013).

One key consideration when designing tactile displays is determining the minimal possible spacing between tactors on a given body part before their loci become indistinguishable because of limits on the skin’s processing capacity. Empirical studies have focused on assessing the maximal throughput of the skin with the tactile spatial acuity threshold as the central parameter of interest (e.g., Novich and Eagleman 2015; Sofia and Jones 2013). Here, we focus on haptic resolution for vibrotactile stimulation for the lower thoracic region, since such passive areas are preferable stimulation sites for tactile devices, because active parts like the tongue, feet and hands should be available for other functions (Dakopoulos and Bourbakis 2010; Kristjánsson et al. 2016).

While spatial acuity for static tactile pressure has been investigated extensively (Gibson and Craig 2005; Mancini et al. 2014; Weber 1834; Weinstein 1968), those findings cannot be generalized to vibratory spatial acuity. Firstly, in contrast to pressure stimuli, the vibrotactile signal spreads beyond the limits of the contact area resulting in signal interference between tactors (Cholewiak et al. 2001). Also, pressure stimuli are processed by different mechanoreceptors than vibratory stimuli. There are five types of mechanoreceptors in the lower thoracic region covered by hairy skin. Whereas Merkel disks and Ruffinis endings are slowly adapting receptors responding when their nerve endings are steadily compressed, such as from skin stretching or grasping objects, Pacinian corpuscles and hair follicle receptors are rapidly adapting mechanoreceptors responding to rapid skin indentation and hair motion, such as from vibration (Gardner and Martin 2013). The fifth type has been termed “field units” and this type is rapidly adapting responding to moving stimuli (Olausson et al. 2000). Therefore, tactile spatial acuity measured with pressure stimuli reflects the response of Merkel disks, which are located in superficial layers of the skin, with very small receptive fields (2–10 mm) and a high number of receptors per nerve ending. Spatial acuity measured with vibratory stimuli at higher frequencies of at least 100 Hz, however, primarily reflects the responses of Pacinian corpuscles, which are located in the subcutaneous skin tissue and have much larger and less numerous receptive fields, resulting in lower resolution (Gardner and Martin 2013). The hair follicle receptors respond to frequencies of 80 Hz and lower (Mahns et al. 2006), and are therefore relevant for stimulation with lower vibrational frequencies. Since most haptic communication devices are nowadays equipped with vibratory tactors, studies specifically investigating vibrotactile spatial acuity are required for further development of such devices.

Investigations of vibrotactile spatial acuity are complicated by the nature of vibrotactile stimulation, causing challenges for across-study comparisons. Apart from the tactor characteristics themselves, such as contact area size (Morioka et al. 2008), spatial acuity estimates are probably influenced by the physical characteristics of vibrotactile signals, which can vary in waveform, amplitude (acceleration), and frequency. While the sense of touch is relatively insensitive to waveform changes in vibrotactile signals of 100–300 Hz (Bensmaia and Hollins 2000), the threshold for detecting the amplitude of a vibratory stimulus varies over the torso, with the lowest threshold on the sternum, of 3.8 µm at 100 Hz, and the highest threshold in the abdominal and gluteal regions (27–29 µm at 100 Hz; Jones et al. 2006). Human frequency sensitivity ranges from 0.4 Hz to more than 500 Hz (Bolanowski et al. 1994). Mahns et al. (2006) found that cutaneous local anesthesia of hairy skin impaired the detection of low frequencies (20–50 Hz), while it had little effect on vibrotactile detection at high frequencies (100–200 Hz). This suggests that the detection of tactors in tactile displays, usually operating at 50–300 Hz (Mortimer et al. 2007), depends on deep receptors, like the Pacinian corpuscles. Additionally, the perception of these properties is not orthogonal: for instance, Morley and Rowe (1990) found that most participants perceived an increase in frequency when the amplitude was increased, even though the frequency of the vibratory stimulation was kept constant.

One basis for understanding the influences of vibratory stimulation involves physical measures to quantify the skin’s viscoelastic properties in response to dynamic mechanical perturbation. Tactor activation creates a surface wave that causes the vibrotactile signal to spread beyond the tactor area (Cholewiak et al. 2001). Franke (1951) used stroboscopic light to determine the amplitude of the surface wave created by vibrotactile stimulation, finding that it decreased in inverse proportion to the travel distance squared (1/d^2^). Boyer et al. (2007) found that the characteristics of surface waves do not depend on the frequency of the vibrotactile signal, but rather on the physical properties of the skin (stiffness and damping effects due to underlying tissue), which greatly varies with body location (Liang and Boppart 2010). Note that in the previously described studies, a custom-made apparatus, such as mechanical wave drivers, was used, and the rigid shell that is usually part of commercially available tactors, such as those used here, might possibly attenuate resulting surface waves. Sofia and Jones (2013) placed four tactors on the palm, forearm and thigh to measure the surface waves resulting from vibrotactile stimulation. They found waves of significantly higher frequency and lower vibration amplitude on glabrous (palm) than hairy skin (thigh, forearm), and that most wave attenuation occurs within the first 8 mm around a tactor, but vibrations were still detectable at a distance of 24 mm, which could mean that tactor spacing should be at least 24 mm. It is also worth noting that not much is known about the propagation of sub-surface waves, although they are likely relevant for vibrotactile perception since Pacinian corpuscles are located in the subcutaneous skin tissue.

It is also important to take neural and cognitive processes involved in tactile perception into account during assessment of vibrotactile acuity, and any assessments must therefore be accompanied by behavioral experiments. A number of psychophysical studies have addressed optimal tactor spacing and different methods have resulted in different estimates of tactile resolution at given body sites. For example, the two-point threshold (2PT) measures the minimum distance for two simultaneously presented stimuli to be distinguished (Sofia and Jones 2013). The 2PT is not suitable for vibrating stimuli, however, since the decision whether one or two tactors are activated can be cued by additive tactor intensities. Alternatively, absolute point localization (aPL) involves determining how accurately a single stimulation point within a defined array of tactors can be located (Cholewiak and McGrath 2006; Lindeman and Yanagida 2003). Here, we use relative point localization (rPL), which involves assessing the minimum distance required to determine the location of a second stimulus relative to the first, presented successively.

Eskildsen et al. (1969) tested successive presentation using a row of five tactors with varying tactor distance on participant’s backs. They found a mean threshold of 10 mm in the thoracic region. Furthermore, van Erp et al. (2005) measured relative spatial acuity by placing 14 (and 11) tactors in horizontal (and vertical) arrays on the back and abdomen, finding uniform tactile acuity across the torso of 20–30 mm, except for arrays located on the body midline, where acuity was approximately 10 mm. Novich and Eagleman (2015) reported surprisingly low tactile acuity when testing an array of 3 × 3 eccentric rotating mass tactors (size: 25 mm) on the back, and argued that these tactors need to be at least 40 mm apart. In our previous work (Jóhannesson et al. 2017), we assessed relative spatial acuity on the lower back with a 3 × 3 array of coin cell eccentric rotating mass motors (10 mm diameter). Our results suggested that these tactors can be differentiated when placed only 13 mm apart (center-to-center) and therefore can be mounted as close as physically possible. Finally, spatiotemporal interactions may also play a role. When two stimuli are presented with relatively short intervals, inter-tactor distance is more likely to be underestimated (Cholewiak 1999).

Taken together, the results of studies measuring vibrotactile spatial acuity are mixed, reflecting the complex nature of vibrotactile stimulation. While some of these factors are commonly acknowledged and considered in psychophysical experiments (e.g., the effect of body area, glabrous vs. hairy skin, choice of paradigm, spatiotemporal interactions), the effects of the chosen tactor type remain unknown. Tactors differ in frequency, amplitude (acceleration), contact area and surface wave, so results for one tactor type may not generalize to another. While certain standards for tactile pressure stimuli have been established (using e.g., von Frey filaments, Cody et al. 2008) enabling comparison across studies, the same is not true for vibrotactile stimuli. In studies of vibrotactile spatial acuity, apparatus description often lacks sufficient technical detail to ensure comparability and replication. Neither are the tactor type characteristics specified, nor are interpretations limited to the tested tactor type. To the best of our knowledge, there are no psychophysical studies specifically investigating differences in outcome for relative vibrotactile acuity measurements with different tactor types.

Additionally, measurements of vibrotactile spatial acuity may depend on presentation direction. Tactile anisotropies (direction dependencies) have been extensively studied for pressure stimuli (Gibson and Craig 2005; Wheat and Goodwin 2000; Wong et al. 1974), revealing higher horizontal than vertical acuity (Lechelt 1988). Cody et al. (2008) investigated localization precision on the upper limb and found acuity to be greater when tactile pressure stimuli were presented along the traverse axis (crossing the arm) than for the longitudinal axis (along the arm). As discussed, however, results on pressure stimuli should not be generalized to vibrotactile perception. Van Erp (2005) found no directional dependency in vibrotactile acuity with a tactor spacing of 20 mm on the torso. Other results with vibratory stimuli, however, reveal sensitivity anisotropies along the skin, where for most sites (except the fingertips) the sensitivity is significantly higher in the medial–lateral direction (e.g., collarbone toward shoulders) than the proximal–distal direction (e.g., wrist towards elbow). Sofia and Jones (2013) placed an array of vibrating motors on the hands, arms and thighs of participants, finding that they were able to identify the correct activation column (medial–lateral) nearly twice as often as the correct row (distal–proximal). A number of neural mechanisms connected with anisotropies have been suggested (Gibson and Craig 2005). Differences in surface wave propagation of vibration along the skin depending on skin stiffness, e.g., due to the type of underlying tissue may also play a role (Sofia and Jones 2013).

Another factor influencing tactile spatial acuity measurements are anchor points, such as the wrist or elbow (Boring 1942). Studies on pressure stimuli suggest that these body landmarks may act as reference points for tactile localization, with localization accuracy highest in the region of joints (Cody et al. 2008) and mislocalization errors occurring in the vicinity of the nearest joint (Margolis and Longo 2015; Medina et al. 2018). For vibrotactile stimulation, Cholewiak and Collins (2003) demonstrated that localization accuracy along the forearm was a direct function of its proximity to the wrist or elbow. The same effect was found for the body midline, with higher vibrotactile localization accuracy at the spine and navel (Cholewiak et al. 2004; Van Erp et al. 2005). Cholewiak et al. (2004) suggest that the body midline has a bilateral cortical representation and might serve as a physical anchor for an internal egocentric coordinate frame.

### Current goals

Our aim was to systematically measure discrimination accuracy for vibrotactile stimulation in the lower thoracic region using relative point localization (rPL) and to identify possible influencing factors on vibrotactile discrimination. Firstly, we assessed the accuracy that can be obtained with each of the three selected tactor types to determine the effect of tactor type on vibrotactile spatial acuity measurements. Furthermore, we systematically assessed the influence of the direction of stimulus presentation. Based on previous evidence for tactile anisotropies, we expected higher vibrotactile spatial acuity for horizontal (medial–lateral) than vertical (proximal–distal) stimulus presentation.

## Method

### Participants

Seventeen students at the University of Iceland participated (12 females, aged between 20 and 26 years, *M* = 23.12, SD = 1.32), receiving course credit for participation. They gave written informed consent before the experiment started and were naïve about the purpose of the study. The experiments were approved by the National Bioethical Committee of Iceland (VSN-15-107).

### Apparatus

#### Mounting structure–vibro-sponge

Each of the three tactor types tested was placed on a separate mounting structure resulting in three devices referred to as vibro-sponges. One vibro-sponge consisted of a 4 × 4 array of the same type of tactor that was glued to an approx. 15 cm-thick layer of foam, where the central part of the device, which covered the participant’s spine, consisted of 4 additional centimeters of foam, to ensure good fit. The foam was mounted on a plastic frame that contained an electronics board, battery and a charger circuit. Custom software written in PsychoPy (Peirce 2009) controlled stimulus presentation. During the experiment, one vibro-sponge at a time was placed centrally in the participant’s lower thoracic region, fastened with straps.

We tested two center-to-center inter-tactor distances (for simplicity, from here on referred to as “distance”) for each tactor type. The experiment was therefore conducted in two sessions where the apparatus was adjusted accordingly in between. The initial distance was based on previous work (Jóhannesson et al. 2017). For the second session, the distance for each tactor type was decreased to the closest distance physically possible. The distance of the tactors therefore varied across sessions depending on the motor type, but was constant for each vibro-sponge within sessions.

#### Normal rotation eccentric rotating mass motors (N ERMs)

**Fig. 1:**
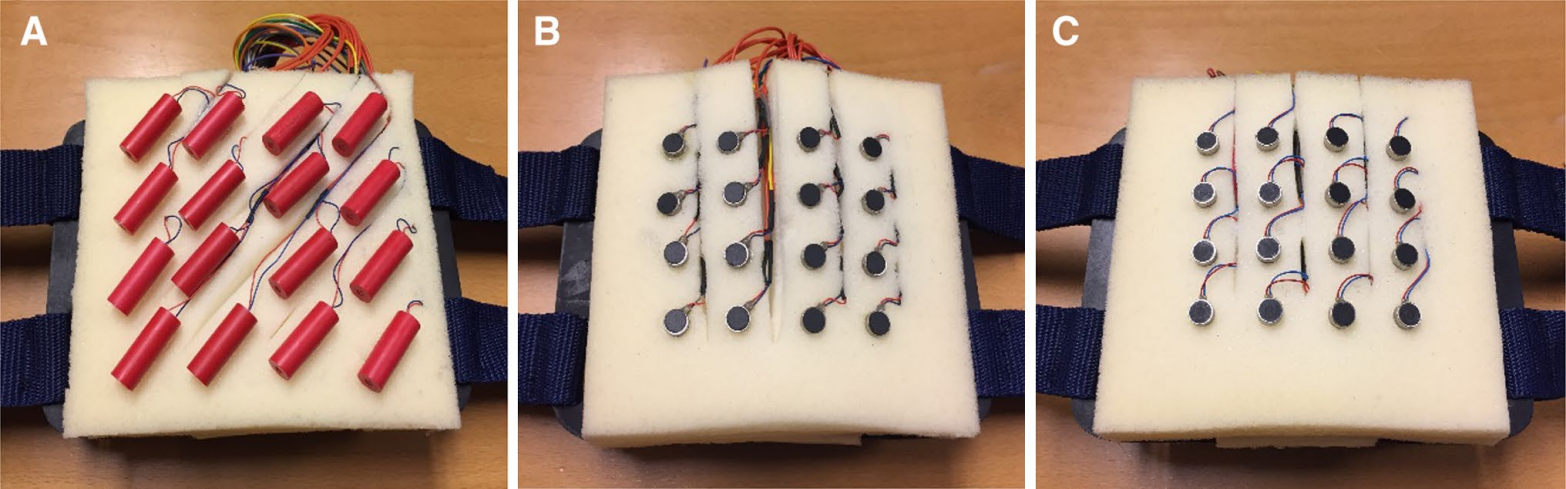
**a** Vibro-sponge I with a 4 × 4 array of normally rotating eccentric rotating mass motors (N ERMs) placed at 25 mm center-to-center (c/c) distance. **b** Vibro-sponge II with a 4 × 4 array of parallel rotating ERM motors (P ERMs) placed at 20 mm c/c distance. **c** Vibro-sponge III with a 4 × 4 array of linear resonant actuators (LRAs) placed at 20 mm c/c distance

Vibro-sponge I comprised 16 eccentric rotating mass motors arranged in a 4 × 4 array, covered by a cylindrically shaped plastic case with body diameter = 8.7 mm and length = 25 mm (model #307-103, Precision 2018a; Fig. 1a). Their rotating mass creates vibration normal to the surface of the skin, which is why they are referred to as N ERMs in the following. Controlled by a simple Darlington driver, the N ERMs were run at half their operating voltage, 2 V (DC), resulting in a vibration frequency of 170 Hz, a current of 65 mA and an acceleration of 4.0 G, as provided by the manufacturer. To determine the specific load frequency for the experimental setup, vibro-sponge I was placed in the lower back and firmly strapped around the waist like in the experiment. The frequency of each individual tactor was analyzed with real-time fast Fourier transform analysis (Advanced Spectrum Analyzer PRO). The results show that the load frequencies ranged between 55 and 67 Hz, with an average of 62 Hz. In the first session, the tactor distance was 25 mm, and 20 mm in the second (the closest distance physically possible).

#### Parallel rotation eccentric rotating mass motors (P ERMs)

Vibro-sponge II comprised 16 P ERM motors mounted in a 4 × 4 array (Fig. 1b). The rotating mass rotates in-plane, creating a vibration parallel to the skin‘s surface, and they are therefore referred to as P ERMs. The P ERMs were controlled by a simple Darlington driver, had a body diameter of 8 mm and height 3 mm, and run on 4 V DC with a frequency of 230 Hz and 1.0 G acceleration (comparable to #308-100, Precision 2018b). The load frequency of the P ERM tactors was assessed and analyzed in the same way as described for the N ERMs. The results show that the load frequency ranged between 126 and 143 Hz, with an average of 132 Hz. This motor type is the most common one in haptic applications as it has a reasonable cost–benefit ratio and is easy to handle. In the first session the tactor distance was 20 mm, and 10 mm in the second (the closest distance physically possible).

#### Linear resonant actuators (LRAs)

Vibro-sponge III contained 16 linear resonant actuators (LRAs) in a coin-shaped metal body, body diameter = 8 mm and height = 3.25 mm, mounted in a 4 × 4 array (Fig. 1c). The mechanical vibration differs from the other two tactors: instead of a rotating motor, a vertically oscillating membrane causes the vibration. Therefore, LRAs can be controlled very precisely with minimal onset and offset delays, and, unlike for the ERM tactors, amplitude and frequency can be altered independently. A Texas Instruments DRV2605L haptic controller was used to change DC to AC. The LRAs were run with an operating voltage of 4 V at a frequency of 235 Hz, with a current of 65 mA and an acceleration of 1.4 G (as provided by the manufacturer, model #C08-001, Precision 2018c). The results of the load frequency analysis (assessed in the same way as for the two ERM tactor types) show that it ranged between 240 and 281 Hz (average = 263 Hz). These LRAs have the same diameter as the P ERMs in vibro-sponge II. In the first session, the tactor distance was 20 mm, and 10 mm in the second session (the closest distance physically possible).

### Paradigm

We used the rPL method, where two tactile stimuli are presented successively on each trial (Weinstein 1968). There were two parts. For the horizontal direction, participants judged whether the second tactor activation was to the left or right of the first tactor activation, or whether it was in the same location (3-alternative forced choice task, 3AFC). Observers responded on a standard keyboard with the left and right arrow keys, if they thought that there had been a location shift, and the space bar if the second tactor activation was perceived in the same location as the first. The procedure was similar for the vertical direction (with the up and down arrow keys). The tactors were turned on for 200 ms with an inter-stimulus interval of 50 ms. The inter-trial interval varied randomly between 1100 and 1700 ms in 100 ms steps. The location of the first tactor, and whether the second tactor was to the left (up), right (down) or in the same location as the first, was randomly determined. Note that the initial tactor always had two adjacent tactors, so a potential subsequent activation could occur on either side. The order of each complete block of trials (which was repeated six times for each motor type and inter-tactor distance) was balanced for the 3AFC answer possibilities and involved 56 trials of each of the three answer types in random order.

### Procedure

The experiment was conducted in two sessions since the equipment had to be adjusted (see “Apparatus” section). Between sessions, distance was decreased, from 25 mm (session 1) to 20 mm (session 2) for the N ERMs, and 20 mm (session 1) and 10 mm (session 2) for the P ERMs and LRAs. Apart from the adjusted distance, both sessions for each tactor type were identical.

After signing informed consent, participants heard an explanation of the 3AFC rPL task. They were subsequently outfitted with the first of the three vibro-sponges, which was placed centrally in the lower thoracic region of the participants’ backs, on top of a thin layer of the participants’ clothes. Participants wore headphones playing white noise during the experiment to mask the sound of the tactors. With the first vibro-sponge, the participants performed the rPL task for one direction, followed by the other. Subsequently, the first vibro-sponge was replaced by the second and the participants repeated the rPL task for both directions, followed by the third vibro-sponge. The order of tactor types and stimulus direction was randomized for each condition. All in all, the procedure took about 1 h for each session.

### Statistical analyses

Balanced design repeated measure ANOVAs were conducted in R (R Core Team 2015) to assess the effects of inter-tactor distance, tactor type and presentation direction (vertical vs. horizontal) on accuracy, while including all possible factors for each level of comparison and reporting *generalized eta-squared statistics* (*η*_G_^2^) as measures of effect size. To determine how close the tactors could be placed while still perceived in separate locations, we conducted one-sample *t* tests assessing whether accuracy significantly differed from chance (0.33) for each tactor type and inter-tactor distance (with Bonferroni-corrected *p* values).

To analyze the effect of the spine as anchor point, the tactor array was divided into spine area and peripheral area as follows (with columns counted from left to right): all stimulus combinations for both directions occurring within the two central columns of the array (2–3) were allocated to the spine area. All horizontal stimuli combinations within the two peripheral columns (within 1–2, and within 3–4), and all vertical stimuli combinations within the outermost columns 1 and 4, were allocated to the peripheral area. An ANOVA assessed the effect of spine area vs. peripheral areas on accuracy, while taking all tactor type and inter-tactor distance conditions into account that would surpass chance-level accuracy.

## Results

**Fig. 2:**
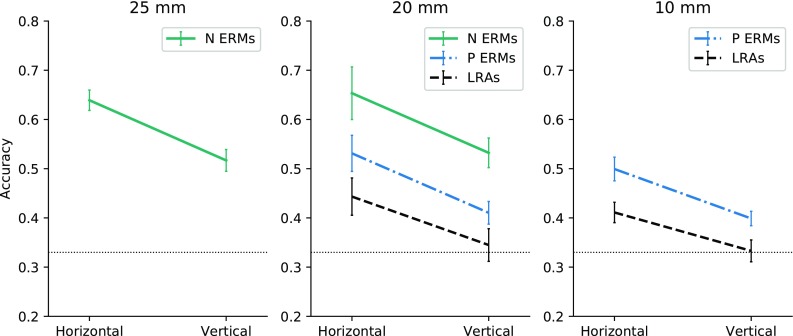
Accuracy plotted by inter-tactor distance (25 mm, 20 mm, 10 mm), direction of stimulus presentation (horizontal vs. vertical), and the three tested tactor types. Note that the variables on the *x*-axis are discrete and the lines are meant to visually connect the conditions. The dotted horizontal line represents the chance level (0.33), and the error bars show the standard error of the mean (SEM)

Figure 2 shows the mean accuracy rates for the three different inter-tactor distances, tested in the experiments, as a function of tactor type and presentation direction.

All accuracy rates obtained with both ERM tactor types were significantly above chance level (0.33) in all conditions. Accuracy rates obtained with the LRAs were significantly above chance when the stimuli were presented horizontally at both 20 mm and 10 mm, but not when presented vertically. For the N ERMs, the difference in accuracy between the 25 mm and 20 mm distance was neither significant for horizontal (*t*(16) = − 0.28, *p* = 0.786) nor vertical presentation (*t*(16) = − 0.39, *p* = 0.699). For the P ERMs, there was no significant change in accuracy between 20 mm and 10 mm, for horizontal (*t*(16) = 1.14, *p* = 0.270) or vertical presentation (*t*(16) = 0.48, *p* = 0.635), and the same was true for the LRAs (horizontal, *t*(16) = 1.02, *p* = 0.321; vertical, *t*(16) = 0.30, *p* = 0.771).

### Tactor type

Figure 2 illustrates that accuracy rates vary greatly by tactor type across all conditions. At the 20 mm inter-tactor distance, we found a highly significant main effect of motor type on accuracy (*F*(2, 32) = 27.99, *p* < 0.001, *η*_G_^2^ = 0.24), but no interaction between the main effects of motor type and presentation direction (*F*(2, 32) = 0.24, *p* = 0.788, for more, see results in “Tactile anisotropy”). Pairwise comparisons revealed significantly higher accuracy for the N ERMs than the P ERMs, with a mean difference of 0.122 (*p* = 0.001, 95% CI 0.050–0.194), and higher accuracy for the N ERMs than LRAs (mean difference of 0.20; *p* < 0.001, 95% CI 0.123–0.274). Further, accuracy rates for the P ERMs were significantly higher than for the LRAs (mean difference of 0.077; *p* = 0.023, 95% CI 0.010–0.144). The same pattern was found for the 10 mm inter-tactor distance, with a highly significant main effect of motor type on accuracy (*F*(1,16) = 25.60, *p* < .001, *η*_G_^2^ = 0.18), but no interaction between the main effects of motor type and presentation direction (*F*(1,16) = 0.50, *p* = 0.489, see further results in “Tactile anisotropy”). Independently of presentation direction, the accuracy rates for the P ERMs were significantly higher than for the LRAs with a mean difference of 0.077 (95% CI 0.045–0.109).

### Tactile anisotropy

Figure 2 suggests that there was a strong spatial anisotropy in vibrotactile perception. The accuracy was higher for horizontal than vertical presentation direction, consistently across tactor types and distances. For the N ERMs (including both inter-tactor distances, 25 mm and 20 mm), there was a highly significant effect of direction (*F*(1,16) = 11.68, *p* = 0.004, *η*_G_^2^ = 0.09), but no interaction between the main effects of distance and direction (*F*(1,16) = 0.00, *p* = .984). For the N ERMs, horizontal accuracy was on average 0.12 higher than vertical accuracy (95% CI 0.066–0.095).

At the 20 mm inter-tactor distance, we could test the main effect of presentation direction for all three tactor types. There was a highly significant main effect of presentation direction (*F*(1,16) = 24.18, *p* < 0.001, *η*_G_^2^ = 0.13), with no interaction between direction and tactor type (*F*(1,16) = 0.24, *p* = 0.788). Across all three tactor types, horizontal accuracy was on average 0.11 higher than vertical accuracy (95% CI 0.065–0.162).

Finally, for the 10 mm inter-tactor distance, there was a significant main effect of direction (*F*(1,16) = 13.17, *p* = 0.002, *η*_G_^2^ = 0.23), independent of tactor type (no interaction between direction and tactor type; *F*(1,16) = 0.50, *p* = 0.489). Across both tactor types, horizontal accuracy was on average 0.09 higher than vertical accuracy (95% CI 0.037–0.141).

### Spine as anchor point

We found a significant main effect of the spine area vs. peripheral areas (*F*(1,16) = 6.43, *p* = 0.022, *η*_G_^2^ = 0.06), a significant main effect of presentation direction (*F*(1,16) = 17.23, *p* < 0.001, *η*_G_^2^ = 0.11), and a significant interaction between the two (*F*(1,16) = 8.84, *p* = 0.009, *η*_G_^2^ = 0.09), while excluding the vertical LRA conditions that did not surpass chance-level accuracy. When vibrotactile stimuli were presented within the spine area, horizontal accuracy was on average 0.15 lower (*M* = 0.48, SD = 0.186; 95% CI − 0.256 to − 0.036) than peripheral to the spine (*M* = 0.63, SD = 0.115; *t*(16) = − 2.81, *p* = 0.013). Vertical accuracy did, however, not differ between stimulation in the spine (*M* = 0.47, SD = 0.109) and peripheral areas (*M* = 0.46, SD = 0.103); *t*(16) = 1.26, *p* = 0.225.

## Discussion

Increased demand for advanced tactile equipment along with effective haptic languages to convey information calls for basic psychophysical investigations of mechanisms underlying the sense of touch. Assessing spatial acuity will contribute to more efficient tactile applications, for example by determining how closely tactors can be placed for better information transmission. We assessed spatial acuity for vibrotactile stimulation in the lower thoracic region for three different tactor types at two inter-tactor distances, for vertical and horizontal presentation, and compared accuracy in the spine area with the peripheral area. Our main incentives were to gain increased understanding of vibrotactile sensitivity for different tactor types, with the aim of raising awareness of potential differences in outcome when different tactor types are used for vibrotactile spatial acuity studies, and, but also to formulate guidelines for the design of tactile displays.

### Tactor type

Our results indicate that vibrotactile discrimination accuracy differs substantially by tactor type with higher accuracy for the N ERMs than for the other two tactor types, and higher accuracy for the P ERMs than the LRAs. The findings are mostly consistent with the specific characteristics of each tactor type. Due to their cylindrical shape, the contact area of the N ERMs depends on how firmly they are pressed against the skin, varying between 125 and 250 mm^2^. Their contact area is 2.5–5 times larger than those of the other two tactors (50.24 mm^2^), and larger contact areas have been found to produce higher sensitivity (Morioka et al. 2008). The force, as related to the perceived intensity of a tactor, depends on the interaction between mass, frequency and acceleration, whereby frequency and acceleration reinforce one another (Bolanowski et al. 1994; Morley and Rowe 1990). In line with the results, the mass of the N ERMs (4.6 G) was about four times higher than that of the P ERMs (0.8 G) and LRAs (0.95 G), and the acceleration of the N ERMs (4.0 G) was highest, four times higher than that of the P ERMs (1.0 G) and about three times higher than that of the LRAs (1.4 G). The N ERMs run at a lower load frequency than the P ERMs (62 Hz vs. 132 Hz). Note, however, that the effects of frequency variation on localization accuracy are typically small (Cholewiak et al. 2001; Cholewiak and McGrath 2006). Whether the frequency–acceleration relation (Bolanowski et al. 1994; Morley and Rowe 1990) increases spatial acuity is not entirely clear. For instance, higher acceleration results in stronger surface waves, travelling further from the origin (Franke 1951), which should decrease spatial acuity. Surprisingly, accuracy was lowest for the LRAs, although they can be controlled most precisely, have a similar contact area and mass as the P ERMs, a slightly higher acceleration than the P ERMs, and their load frequency is 2–4 times higher (263 Hz) than of the other tactors. The latter is in line with findings that frequency does not have a strong effect on vibrotactile spatial acuity (Cholewiak et al. 2001). Instead, the difference in accuracy might reflect the way the vibration is generated. The N ERMs generate a complex vibration pattern with rotation on both ends of the tactor causing both perpendicular motion (toward and away from the skin’s plane) and motion parallel to the skin’s plane. Such multiple vibration stimulation might increase perceived intensity and thereby facilitate discrimination perception. LRAs, on the other hand, create force by a magnetic mass attached to a spring and driven by a voice coil (Precision 2018d), resulting in vibration exclusively directed perpendicular to the skin’s surface, which may confine the vibrations and cause smaller surface waves. While lesser vibration spread should improve localization, it might also lower perceived intensity. Azadi and Jones (2014) found that, if put under load, LRAs tend to show a stronger decrease of mechanical input delivered to the skin than other tactor types, possibly affecting the user’s ability to detect the tactile input. In fact, participants in our study reported that it was difficult to discern differences between the LRAs. Future studies should focus on investigating the nature of vibration created by LRAs compared to eccentric mass-based tactors.

When relating the current results to previous studies, our findings for the P ERMs complement the results of Jóhannesson et al. (2017), who found that P ERMs (10 mm diameter), in the same rPL task could be placed as close as physically possible (13 mm c/c) leading to 64% discrimination accuracy. With smaller P ERMs in the current study (8 mm diameter), the inter-tactor distance could be decreased to 10 mm c/c, but even though participants were still able to discriminate two adjacent tactors, accuracy dropped to 45%. Overall, the relatively low accuracy found for P ERMs and LRAs seems to accord well with their small size (as related to force), and the very small tactor distance of only 2–3 mm in between them (10 mm center-to-center), indicating approximation of the threshold of vibrotactile discrimination acuity. Notably, however, the accuracy for the N ERMs was higher than in the results of Novich and Eagleman (2015), who tested the same N ERMs in the same body area using the 2PT method with either spatial stimuli (single motor) or spatiotemporal stimuli (sweeps of two motors). They reported that accuracy was only higher than chance at a tactor distance of 40 mm. In our study, we constrained the N ERMs to 62 Hz load frequency because preliminary tests revealed that participants felt uncomfortable when we ran them at 120 Hz, or higher. In our 3AFC task using the rPL method and spatial stimuli, the accuracy for N ERMs was higher than chance at a 20 mm c/c distance, with accuracy rates of 65% (53%) for horizontal (vertical) presentation. A possible explanation for the lower accuracy in Novich and Eagleman (2015) is that the tactors were run at a high frequency (340 Hz) and acceleration (> 8.0 G, assumingly spec values), which may have created far-traveling surface waves (Franke 1951) that blurred the tactile signal. Even though frequency seems to have a small effect on spatial acuity (Cholewiak et al. 2001; Cholewiak and McGrath 2006), this probably does not apply here as the frequency tested in these studies ranged from 80 to 250 Hz. Another reason for the low accuracy in Novich and Eagleman (2015) might be the paradigm. They asked participants to choose whether they perceived one or two stimuli, even though “one” was never presented. This may have led participants to choose “one” because they expected “one” to be a required answer at some point which would underestimate the accuracy for the N ERMs.

### Tactile anisotropy

Tactile acuity was higher for horizontal (medial–lateral) than vertical presentation (proximal–distal). Across all conditions, participants performed better when differentiating between columns than rows. Similar and possibly related tactile anisotropies have been found for pressure stimuli in various settings, for instance, for gap detection tasks (Gibson and Craig 2005), absolute localization (Margolis and Longo 2015; Medina et al. 2018), or when participants judged inter-stimulus distances (Longo and Haggard 2011). For vibrotactile stimulation, however, the results are mixed, with some studies finding anisotropies (Sofia and Jones 2013) and others not (Van Erp 2005). This vibrotactile anisotropy has implications both for tactile acuity measurements and for designing tactile displays.

According to the results of Gibson and Craig (2005), the direction and degree of anisotropy is inconsistent across locations suggesting influences of a complex network of variables. Liang and Boppart (2010) quantified the viscoelastic properties of human skin, testing orientations parallel or orthogonal to the Langer’s lines (topological lines corresponding to the natural orientation of collagen fibers in the dermis; Langer 1978) and reported that skin stiffness is anisotropic, depending on the orientation of Langer’s lines. Skin stiffness is more parallel to the Langer’s lines than in the orthogonal direction. The surface wave caused by vibrating stimuli could be more strongly inhibited along the Langer’s lines, facilitating differentiation between two vibrating stimuli. Given that Langer’s lines in the lower thoracic region run medial–lateral, differentiating between columns in a tactile display (ventral–lateral stimulation) should be more accurate than differentiating between rows (dorsal–proximal). However, Liang and Boppart (2010) only found this for high frequencies (600 Hz), while for frequencies of 50 Hz, measurements of skin stiffness in both directions were comparable (as in Sofia and Jones 2013). Even though skin anisotropy may partly be related to stimulus orientation with respect to Langer’s lines and, in the case of hands, to skin ridges (Vega-Bermudez and Johnson 2004; Wheat and Goodwin 2000), other mechanisms appear to be involved.

It has been suggested that the receptive fields of primary afferents and their higher-order neurons may be oval shaped and elongated along the proximal–distal axis (Stevens and Patterson 1995; Cody et al. 2008). Even though there is no evidence for distortions in the shape of the receptive fields of afferent fibers, there are anisotropies in the shape of receptive fields of neurons in the spinal cord and somatosensory cortex (Brown et al. 1975; Alloway et al. 1989). Medina et al. (2018) suggested that the directional bias commonly found in absolute localization tasks for touch (Margolis and Longo 2015) may reflect distortions of a supramodal representation of the skin surface and demonstrated that the directional bias can be modulated by gaze direction. Additionally, attentional mechanisms and the enhancement of resolution at anchor points (joints, spine, see discussion below) have been suggested as possible variables modulating tactile anisotropy (Cody et al. 2008; Medina et al. 2018).

### The spine as anchor point

Overall, localization accuracy was lower in the spine area than more peripherally. Vibrotactile stimuli directly located at/or crossing the body midline were more poorly localized than stimuli along the spine. This effect only involved the horizontal presentation direction, however, which may reflect a floor effect due to the lower overall vertical accuracy. These results contradict the common finding of increased tactile acuity with closer distance to anchor points (Boring 1942; Cody et al. 2008; Cholewiak and Collins 2003; Cholewiak et al. 2004). It is worth noting that although both body midline and limb landmarks are usually subsumed under the term of anchor points, the results of studies on limb areas (Boring 1942; Cody et al. 2008; Cholewiak and Collins 2003) might not be directly applicable to the body midline. Wrist and elbow are often referred to as points of mobility (Boring 1942), and Cody et al. (2008) have argued that increased tactile acuity may contribute to improved proprioceptive guidance of active wrist movements. The spine cannot serve the same function and, although there is evidence for a similar effect of higher acuity for the body midline, other neurocognitive mechanisms might underlie this finding (Cholewiak et al. 2004).

A probable explanation is the increased spread of vibration along the dorsal vertebra of the backbone, a key difference between vibrotactile and tactile studies. The characteristics of surface waves spreading from a vibrating source depend strongly on the physical properties of the skin and its underlying tissue (Boyer et al. 2007; Liang and Boppart 2010). We ensured that the tactors were firmly pressed against the lower thoracic area. Due to the lack of underlying damping tissue between the tactors and dorsal vertebra, vibrotactile stimulation probably spread further beyond the tactors that were located directly at the backbone than alongside of it. Cholewiak et al. (2004) found higher localization accuracy for vibratory stimuli at the spine, which appears to contradict our findings. But note that they used substantially bigger tactors and much higher inter-tactor distances (at least 64 mm), with one of their tactors located at the spine, covering the whole dorsal vertebra. Here, three tactors (8 mm) were placed within the same area, and differences between 10 mm inter-tactor distances were reported. This increased sensitivity allowed for more fine-grained assessment and may therefore yield different results. Further studies will have to explore the detailed characteristic of the localization distortion, for instance by precisely mapping the mislocalization errors in the spine area, and find ways of attenuating the spread of the vibration.

### Study limitations

It is important to emphasize that the vibrotactile discrimination accuracy rates are limited to the lower thoracic region. Tactile spatial acuity differs greatly by body location due to variations in mechanoreceptor density, which is higher on glabrous than hairy skin (Bolanowski et al. 1994), and lower spatial acuity of passive (e.g., torso, arms and legs) than active body areas (Weinstein 1968). We focused on spatial acuity of the lower thoracic region since such passive areas are better suited to tactile presentation than active parts like the tongue, feet and hands, as they need to be available for performing other functions (Kristjánsson et al. 2016). The lower tactile resolution of passive areas like the torso can be compensated for by the larger skin area that can be stimulated.

The reported tactor acceleration reflects information from the manufacturer and only applies to operation without load (referred to as spec values). When tactors are pressed against the skin (e.g., with straps or elastic fabric, as in our experiment and as common for tactile applications), their characteristics change. Hence, the load frequency for each tactor type was assessed specifically for the experimental setup, showing that, when being exposed to the same pressure, the frequency of both ERM tactor types decreased by approx. 100 Hz, while the frequency of LRAs increased by 28 Hz. Compressing the LRAs under load modifies the resting position of the internal spring, which leads them to vibrate at higher frequency. Azadi and Jones (2014) further found a higher resonant frequency when the LRAs were placed on the finger as compared to the forearm, indicating that their resonant frequency depends on the stiffness of the skin they are mounted on. This notable difference between spec and load values, as well as the inconsistency in their change under load condition, emphasizes the importance of reporting load values additionally to spec values. So far, only a few psychophysical studies on vibrotactile spatial acuity involving tactors have reported load characteristics (Azadi and Jones 2014; Cholewiak et al. 2004; Sofia and Jones 2013). Further considerations regarding load values are that there is no standardized way of measuring them, and that load assessment is not feasible in experimental setups with a closed apparatus design (when the tactors are encompassed by a tactile device). Note also that the actual load exerted on each individual tactor can vary across participants and even within the same participant, depending on physique, posture and breathing. As descriptions of apparatus often lack sufficient detail for replication, we recommend discussion of the spec characteristics, supplementing them with available load values and establishing a standardized way of measuring load characteristics to ensure comparability.

Furthermore, the ratio of tactors and area varies across studies. Eskildsen et al. (1969) tested 5 × 1 tactor arrays, van Erp (2005) 14 × 1 and 11 × 1 arrays and van Erp et al. (2005) tested 8 tactors. Lindeman and Yanagida (2003) measured absolute acuity with an array of 3 × 3 tactors with 60 mm spacing finding an accuracy of 84%. Jones and Ray (2008) used an array of 4 × 4 tactors with the same spacing finding an average accuracy across all tactors of 59%. Although the number of tactors differs considerably between these studies, accuracy by distance was similar. Cholewiak et al. (2004) found no consistent effects of tactor number on localization, concluding that the most important factor for localization accuracy is the inter-tactor distance. In line with these findings, the results of Jóhannesson et al. (2017) suggest that decreasing the size of the area of vibrotactile stimulation does not significantly affect the thresholds for relative vibrotactile spatial acuity.

Additionally, results acquired with the relative point localization (rPL) method, as used here, are not directly comparable to other measurement methods, like absolute point localization (aPL, Sofia and Jones 2013) and two-point thresholds (2PT; Weber 1834). Weinstein (1968) found that spatial tactile acuity with the 2PT was two to four times lower than with the aPL, although they were highly correlated. However, the 2PT method cannot be directly applied to vibrating stimuli, since decisions whether one or two tactors are activated can be affected by additive tactor intensity. As discussed above, Novich and Eagleman (2015) introduced a fake stimulation condition to avoid additive intensities when applying the 2PT method with vibrating stimuli, which might lead to an underestimation of spatial acuity. Even though many studies have used aPL (Cholewiak and McGrath 2006; Lindeman and Yanagida 2003; Sofia and Jones 2013), the ability to localize a point of vibrotactile stimulation may not accurately reflect relative spatial acuity (Jones 2011).

All participants were young adults aged from 20 to 26 years, so generalization to older groups requires caution, since vibrotactile acuity decreases with age, especially for high frequencies (Deshpande et al. 2008). Stevens and Patterson (1995) gathered 1478 individual tactile acuity thresholds, finding that tactile acuity decreases by approximately 1% annually. Devices aimed at helping the elderly should therefore be designed with the caveat that we may be overestimating vibrotactile acuity.

## Conclusions

We explored spatial acuity for vibrotactile devices conveying information through touch. This is of high relevance for the design of tactile displays. Our results strongly suggest that the LRAs tested are not an advisable choice for tactile high-resolution displays with dense tactor arrays. Although LRAs seem appealing because frequency and amplitude can be independently controlled (unlike for ERM tactors), the discrimination accuracy for the LRAs does not seem sufficient for any high-resolution tactile display, independent of purpose. It is worth noting, however, that above chance performance as reported here may not be a particularly ambitious goal for conveying information and our aim was not to determine the absolute accuracy for any particular device. The required level of discrimination accuracy strongly depends on the particular goals in each case.

Our results revealed substantial differences between tactor types and show that tactor type can affect measurements of vibrotactile spatial acuity. The comparability of studies using tactors measuring tactile spatial acuity could be considerably improved by providing more detailed information on stimulation and a restrictive evaluation. Hence, we encourage researchers in this field to:

(1) consider including tactor type as experimental condition,

(2) provide detailed technical information on tactors and apparatus to facilitate replication,

(3) discuss differences in tactor type characteristics when comparing vibrotactile spatial acuity measurement results to related work,

(4) emphasize that results obtained with a specific tactor type can only be generalized to other tactor types with great caution.

(5) foster academic debate to establish a standard for the measurement and reports of vibrotactile acuity studies involving tactors.
